# The Shape Shifting Story of Reticulocyte Maturation

**DOI:** 10.3389/fphys.2018.00829

**Published:** 2018-07-11

**Authors:** Elina Ovchynnikova, Francesca Aglialoro, Marieke von Lindern, Emile van den Akker

**Affiliations:** ^1^Department of Hematopoiesis, Sanquin Research, Amsterdam, Netherlands; ^2^Landsteiner Laboratory, Academic Medical Center, University of Amsterdam, Amsterdam, Netherlands

**Keywords:** reticulocytes, erythropoiesis, differentiation, protein sorting, enucleation, maturation

## Abstract

The final steps of erythropoiesis involve unique cellular processes including enucleation and reorganization of membrane proteins and the cytoskeleton to produce biconcave erythrocytes. Surprisingly this process is still poorly understood. *In vitro* erythropoiesis protocols currently produce reticulocytes rather than biconcave erythrocytes. In addition, immortalized lines and iPSC-derived erythroid cell suffer from low enucleation and suboptimal final maturation potential. In light of the increasing prospect to use *in vitro* produced erythrocytes as (personalized) transfusion products or as therapeutic delivery agents, the mechanisms driving this last step of erythropoiesis are in dire need of resolving. Here we review the elusive last steps of reticulocyte maturation with an emphasis on protein sorting during the defining steps of reticulocyte formation during enucleation and maturation.

## Introduction

Production of red blood cells for transfusion can have several distinct benefits for healthcare and patient wellbeing (Douay and Andreu, 2007; Goodnough and Murphy, 2014; Shah et al., 2014; Benedetto et al., 2015). Alloimmunization is one of the major complications of blood transfusion and particularly affects patients who are frequently transfused. Observational studies conducted on random patients who received incidental transfusions demonstrated 1–3% alloimmunization. However, in patients with sickle cell disease (SCD) who receive chronic transfusions this can increase to 8% or even as high as 76% (Campbell-Lee and Kittles, 2014; Alkindi et al., 2017). In severe cases the only option available for the patient is a stem cell transplantation, which carries significant risk on its own. *In vitro* production of transfusable red blood cells can ensure better availability of matched blood, helping in the reduction of alloimmunization and contributing to more efficient treatment of such diseases as hemoglobinopathies and MDS. Apart from possible immune reactions, donor blood can be a source of blood borne diseases such as Hepatitis B and HIV. Produced *in vitro* red blood cells will be safe in terms of infections spreading. Another application for *in vitro* cultured red cells may be as vehicle for therapeutic agents, delivering cargo to specific parts in the body (Muzykantov, 2010; Crossan et al., 2011). Production of cultured red blood cells, however, faces a couple of challenges. First is quantity of production. One unit of blood transfused to the patient contains about 2 trillion red blood cells and annual transfusion need counts up to 90 million units in the world annually (World Health Organisation [WHO], 2017). Currently existing laboratory culture systems of red blood cells are not able to generate satisfactory number of red blood cells in efficient and cost-effective fashion, which need to be improved. Second challenge of red blood cells production is the quality of product. Existing laboratory culture systems give rise to reticulocytes of different stages of maturity using various culture systems and starting material depending or not depending on co-culture with specific stromal cells (Migliaccio et al., 2002, 2010; Giarratana et al., 2005; Leberbauer et al., 2005; Miharada et al., 2006; van den Akker et al., 2010a; Shah et al., 2016; Trakarnsanga et al., 2017). Reticulocytes are immature red blood cells that lost their nucleus but still retain residual RNA. Reticulocytes can perform the main function of red blood cells-oxygen transport. However, they have not yet adopted the unique biconcave shape of mature red blood cells that ensures their stability and flexibility to withstand blood flow shear stress. Luc Douay and coworkers published unique research in which they performed autologous transfusion of ^51^Cr labeled *in vitro* cultured red blood cells to a human volunteer (Giarratana et al., 2011), reporting a reticulocyte half-life of approximately 26 days post injection. The life span of red blood cell is 120 days, which suggests that the *in vitro* cultured reticulocytes exhibit high clearance, possibly due to their low stability and immaturity. Effective use of cultured red blood cells for transfusion purposes may benefit from maturation of reticulocytes to mature erythrocytes, a process that is ill-defined and poorly understood. It is known that after the enucleation process, reticulocytes enter the bloodstream where they complete their maturation into fully functional erythrocytes within 3 days (Chasis et al., 1989). The reticulocyte stage of erythroid differentiation is brief, however, involves an extensive array of changes. During maturation reticulocytes undergo membrane remodeling, lose up to 20% of membrane, eliminate residual organelles and RNA and gain bi-concaveness (Gronowicz et al., 1984). Understanding the processes and factors involved in maturation of reticulocytes is an important step toward development of culture system producing transfusion-ready red blood cells. This review covers recent findings in the process of reticulocyte maturation with an implication in red blood cell production. **Table 1** indicates commonly used abbreviations throughout the review.

**Table 1:** Abbreviations and function.

Acronym	Full name
MDS	Myelodysplastic syndrom
HIV	Human Immunodeficiency Virus
BMP-4	Bone Morphogenetic Protein 4
IL-6	Interleukin 6
TNF-alpha	Tumor Necrosis Factor alpha
INF-gamma	Interferon gamma
VCAM-1	Vascular Cell Adhesion Molecule 1
ICAM-4	Intracellular Adhesion Molecule 4
Gas6	Growth arrest-specific 6
Epo	Erythropoietin
PI3K	Phosphatidylinositol-4,5-bisphosphate 3-kinase
PKB	Protein Kinase B
TAM-receptor	Tyrosine Activation Motif receptor
CAR	Contractile Actomyosine Ring
CRIK	Citron Rho-interacting Kinase
Rho-GTPase	Rho-Guanosine TriPhosphate
MLC2	Myosin regulatory Light Chain 2
HAT	Histone Acetyl Transferase
HDAC	Histone Deacetylase
Gnc5	General control non-derepressible 5
MERTK	Proto-oncogene tyrosine-protein kinase MER
CD34	Cluster of differentiation; transmembrane phosphoglycoprotein
RhAG	Rh-Associated Glycoprotein
GPC	Glycophorin C
EMP	Erythroblast Macrophage Protein
CD147	Basignin
Xk	Kell blood group precursor
Rh	Rhesus
CD47	Integrin Associated Protein
CD49d	Integrin alpha subunit
CD71	Transferrin Receptor 1 (Trf1)
TO	Thiazole Orange
KCC	Potassium Chloride Cotransporter
LPA	Lysophosphatidic Acid
SCD	Sickle Cell Disease
Lu	Lutheran
BCAM	Basal Cell Adhesion Molecule
PKA	Protein Kinase A
GLUT4	Glucose transporter type 4
GPA	Glycophorin A
MVEs	Multivesicular Endosomes
ESCRT	Endosomal sorting complexes required for transport
AQP1	Aquaporin 1
MVB	Multivesicular Body
LC3	Light chain 3b
Rab11	Ras-related protein
LW	Landsteiner-Wiener
CD44	Cell surface glycoprotein

## The Elusive and Ill-Defined Role of Macrophages Within Erythroblastic Islands

**FIGURE 1:**
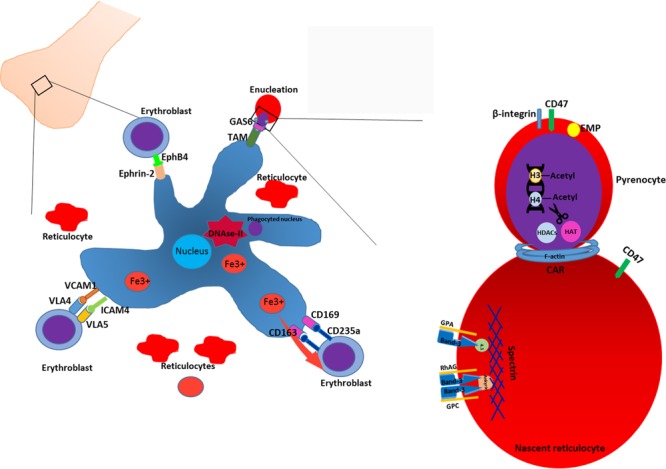
Enucleation and reticulocyte formation in the bone marrow. Erythropoiesis in the bone marrow occurs in structures termed macrophage islands consisting of a central macrophage, surrounded by erythroid cells at different maturation stages and reticulocytes. In order to facilitate the interaction with erythroblasts, macrophage expresses various adhesion molecules such as VCAM1, ICAM4, CD169, CD163. In the erythroid niche, central macrophages play a nursing role for developing erythroblasts. Moreover, macrophage facilitates the enucleation process by phagocytosing and degrading of extruded nuclei through, e.g., interacting with erythroblast via, e.g., GAS6-TAM ligand-receptor tandem. Enucleation of erythroblasts is a complex process which includes condensation of chromatin, deacetylation of histones, formation of CAR and further abscission. Enucleation is coupled to the sorting of unwanted proteins to the pyrenocyte and retention of essential proteins. Generally, cytoskeleton-associated proteins and integral membrane proteins remain in the reticulocytes. Adhesion molecules such as β-integrin, are predominantly sorted to the pyrenocytes (see text).

Terminal erythroid differentiation within bone marrow as well as during ontogeny takes place at specialized multicellular structures called erythroblastic islands (Bessis, 1958; Mohandas and Prenant, 1978) (**Figure 1**). Erythroblastic islands are composed of a central macrophage surrounded by differentiating erythroblasts and reticulocytes. It is unknown whether the primary function of the central macrophage is mainly to engulf and digest the expulsed nuclei, or whether the mutual interaction between the macrophage and the maturing erythroblasts contribute to the erythroid differentiation program and/or macrophage function. Adding to this confusion are the numerous knockouts that interfere with putative interaction partners facilitating the association between erythroid and macrophages that may or may not result in anemia (Sadahira et al., 1995; Hanspal et al., 1998b; Lee et al., 2006; Soni et al., 2006; Heideveld and van den Akker, 2017). Equally confusing is the observation that specific ablation of CD163+ macrophages in mice, although somewhat attenuating erythropoiesis, does not result in anemia (Chow et al., 2011). Differentiating erythroblasts follow distinct morphological changes that define the nomenclature. The early erythroblast stage is characterized by a large cell size, 12–20 μm in diameter, with a nucleus that occupies up to 80% of the cell volume. Nuclei of erythroblasts contain a large number of nucleoli. Basophilic erythroblasts are smaller and characterized by increased condensation of chromatin, combined with basophilic cytoplasm. Polychromatic erythroblasts are characterized by emerging hemoglobinization of the cytoplasm and irregularly condensed nuclei. In orthochromatic erythroblasts hemoglobinization is nearly completed, and nuclei are pyknotic. In this stage the cells do not expel several nuclei expulsion and differentiation to reticulocytes. The concept of erythroblastic islands was introduced by Bessis (1958) who analyzed transmission electron micrographs of bone marrow sections (Bessis, 1958). Bessis hypothesized that central macrophages play a supporting role during erythropoiesis by providing ferritin to the cells and by phagocytosis of expelled nuclei (Bessis and Breton-Gorius, 1962). The hypothesis of Bessis was reinforced by the study of Mohandas on hyper transfused rats (Mohandas and Prenant, 1978) in which erythropoiesis and the number of erythroblast-macrophage clusters is suppressed. Single stimulation of these rats with erythropoietin induced synchronous differentiation of resting erythroblast precursor cells. These experiments allowed to detect the individual clusters of erythroblasts within the bone marrow and link the formation of these multicellular structures to erythropoiesis. Quantitative light and electron microscopy of rat bone marrow sections demonstrated that islands form far from the sinuses accommodate mostly pro-erythroblasts, while the islands situated in the proximity to bone marrow sinuses contained more of differentiating erythroblasts and reticulocytes (Mohandas and Prenant, 1978; Yokoyama et al., 2003). It was proposed by Yokoyama et al. (2003) that erythroblastic islands can migrate toward the sinuses as differentiation of erythroid cells progresses, albeit through unknown mechanisms. This hypothesis, however, requires experimental reinforcement as it is unknown what signals, intrinsic or extrinsic, drive this apparent dynamic process. Co-culture of CD34+ cells with stromal cells or macrophages results in nearly complete enucleation, compared to feeder-free cultures (Giarratana et al., 2005 #729; Fujimi et al., 2008; Lu et al., 2008 #1723). Importantly, other studies have shown that erythroid terminal differentiation to complete enucleation can occur independent of central macrophages and that resulting reticulocytes can mature to biconcave erythrocytes upon injection into the bloodstream (Leberbauer et al., 2005; Ji et al., 2010; van den Akker et al., 2010a; Giarratana et al., 2011). So, although for enucleation and reticulocyte formation macrophages appear to be dispensable, macrophages may provide signals that optimize specific but ill-defined erythroid maturation processes that may be crucial for the eventual reticulocyte stability and function. In addition, specific culture systems/protocols may be more suitable to investigate specific signal transduction and transcriptional programs induced by the interplay between macrophages and erythroid cells and may for instance only manifest upon suboptimal culture conditions. For instance, macrophages within the erythroblastic island regulate erythropoiesis by multiple mechanisms including secretion of soluble factors and cell–cell contact. Macrophages have been shown to secrete BMP4, which can stimulate proliferation of erythroblasts (Millot et al., 2010; Heideveld et al., 2015). Macrophages also support erythropoiesis by secreting ferritin which is later taken up by erythroblasts via transferrin receptor or endocytosis (Meyron-Holtz et al., 1999; Leimberg et al., 2008; Kim et al., 2015). Secretion of IL-6, TNF-α, and INF-γ by macrophages down-regulates erythroid differentiation, while TGF-β suppresses proliferation of early erythroblasts, and enhances differentiation (Zermati et al., 2000; Bohmer, 2004). Direct cell-cell interactions also regulate erythropoiesis within the islands. Several adhesion molecules have been identified that direct contact of erythroblasts with the central macrophage. Emp knockout mice die perinatal due to severe defects in hematopoiesis and have a complete lack of F4/80 erythroblastic island macrophages, whilst erythroid cells display defects in enucleation and undergo increased apoptosis (Hanspal et al., 1998b). Another protein described to mediate erythroid-macrophage interaction and which can be used as a marker of central macrophages is VCAM-1 (Chow et al., 2011). Erythroblasts utilize α4β1 integrin and ICAM4 to bind to VCAM-1 and establish a tight contact with the macrophage and neighboring erythroblasts (Palis, 2016; Seu et al., 2017). Blocking of α4β1integrin with antibodies decreased cell proliferation and increased apoptosis *in vitro* and arrests erythropoiesis at E12 in α4 deficient mice (Sadahira et al., 1995; Lee et al., 2006). Interestingly though VCAM knockout mice do not display any phenotype regarding erythropoiesis, shedding doubt on whether VCAM is the interaction partner of α4β1 integrin (Ulyanova et al., 2016). Erythroblasts themselves can modulate inhibitory signaling from macrophages. Erythropoietin induces the secretion of Gas6 by erythroblasts which enhances the survival response to Epo-receptor signaling through PI3K and PKB-kinase signaling (Angelillo-Scherrer et al., 2008) and may function to facilitate phagocytosis of extruded nuclei through GAS6-dependent TAM-receptor present on central macrophages, which are expressed on human bone marrow and fetal liver macrophages (Toda et al., 2014; Heideveld and van den Akker, 2017). So although specific partners that facilitate the interaction between erythroid cells and macrophages as well as paracrine signaling has been found, the precise role of the central macrophage to the differentiation process of erythroid cells remains ill-defined. Recent phenotypic analysis of central macrophage populations in human bone marrow and fetal liver, together with the development of *in vitro* models now allows for *in vitro* experiments that could shed further light onto this elusive macrostructure (Hom et al., 2015; Belay et al., 2017; Heideveld and van den Akker, 2017; Seu et al., 2017).

## Enucleation

Enucleation of late stage erythroblasts or orthochromatic normoblasts is a unique cellular process that gives rise to reticulocytes [also reviewed in (Migliaccio, 2010)]. The only other cells in the body that undergo enucleation are the cells in the lens of our eyes. Before enucleation occurs, erythroblasts undergo cell cycle arrest, the chromatin condenses and the nucleus locates to the edge of the cell (Keerthivasan et al., 2011). Initially, enucleation was thought to be a specific form of apoptosis, which was based on microscopic detection of karyolysis and partial leakage of nuclear content into the cytoplasm (Simpson and Kling, 1967). In addition, the enucleation process in keratinocytes and fiber lens cells has also been described as a specific mechanism of programmed cell death (Bassnett, 1997; Yamamoto-Tanaka et al., 2014). However, reduction of caspase expression or inhibition of caspase activity demonstrated little to no involvement of caspases in erythroid enucleation (Carlile et al., 2004; Yoshida et al., 2005). Instead, recent evidence supports a model of asymmetric cytokinesis to separate the nucleus from the cell. This implies that the enucleating cell should display a cleavage furrow, the contractile actomyosine ring (CAR) and a stage of completion with abscission (Barr and Gruneberg, 2007) (**Figure 1**).

### The Mechanism of Enucleation

A few players in cytokinesis were shown to play a role in enucleation. Citron Rho-interacting kinase (CRIK), a mitotic kinase known to interact with Rho-GTPases (Magor et al., 2015), regulates the length of astral microtubules and the orientation of the spindle in dividing cells. During enucleation, CRIK participates in the nuclear condensation and is involved in the formation of the CAR, possibly via interaction with MLC2 (Swartz et al., 2017). Another essential factor involved in enucleation is deacetylation of histones H3 and H4. Regulation of the acetylation status of those histones is carried out by enzymes histone acetyl transferase (HAT) and histone deacetylase (HDACs) (Ji et al., 2010). It has been shown that during late stage fetal erythropoiesis in mice Gcn5 and c-Myc are downregulated and histone deacetylase 2 is upregulated, which results in the chromatin condensation and CAR formation (Jayapal et al., 2010). Konstantidinis described the formation of an actin-myosine ring upon initiation of enucleation. Actin-myosin ring contracts and the condensed nucleus is expelled out of the cell together with the thin rim of cytoplasm and plasma membrane to form the pyrenocyte (Konstantinidis et al., 2012). As well as in cytokinesis, the cytoskeleton plays an important role in erythroblast enucleation. Immunofluorescent and microscopic studies demonstrated the condensation of F-actin filaments and formation of CAR can be disrupted by cytochalasin and depends on Rho-GTPase activity and downstream mDia activation (Sawada et al., 1989; Ji et al., 2008). Formation of microtubules plays an essential role in nucleus polarization and extrusion. It has been shown that inhibition of PI3K decreases nuclear polarization efficiency and delays the enucleation in mice (Wang et al., 2012). The mechanism of asymmetric cytokinesis is sustained by a second process that may act concurrently: the formation and trafficking of vesicles during abscission. During this final stage of cytokinesis, vesicles move toward the midbody region, fuse and promote the separation of the daughter cell (Gromley et al., 2005). Vesicles mainly contribute to enucleation by supplying membranes to the progressing tip of the cleavage furrow and thus facilitating the separation of pyrenocyte from reticulocyte. Disruption of vesicle trafficking and inhibition of clathrin expression blocked the enucleation of erythroblasts, suggesting that vesicle trafficking has an essential role in nuclear extrusion process (Keerthivasan et al., 2010). Of note, the eventual phagocytosis of the extruded nuclei occurs via protein-S dependent and MERTK dependent processes (Toda et al., 2014). High enucleation rates have been described for *in vitro* culture systems that initiate from cord blood, fetal or adult CD34+ hematopoietic stem and progenitor cells (Leberbauer et al., 2005; van den Akker et al., 2010a; Giarratana et al., 2011). In contrast, enucleation is generally poor in immortalized cell lines and erythroid cells derived from induced pluripotent stem cells (Lapillonne et al., 2010; Kurita et al., 2013; Trakarnsanga et al., 2017) [reviewed by (Focosi and Amabile, 2017)]. Resolving the fine details of the enucleation process and events leading up to enucleation may facilitate and improve the production of erythrocytes from these immortal sources. This is important as immortalized lines provide a considerable simplification of the *in vitro* erythrocyte production method, can be genetically altered to express specific blood groups or therapeutic cargo and can be standardized.

### Protein Sorting During Enucleation

During erythroblast enucleation, plasma membrane and cytoskeletal proteins undergo reorganization while the nucleus, surrounded by plasma membrane, separates from the reticulocyte. A key aspect of this process is the distribution of (membrane) proteins to the pyrenocyte and/or reticulocytes, which may be through active sorting of specific proteins or through non-specific simple redistribution. By consequence, protein sorting during enucleation determines the protein content of reticulocyte membranes and possibly also intracellular proteins. Cytoskeletal proteins important for erythrocyte function, such as the erythrocyte Spectrins, Ankyrin, and protein 4.1, remain within the nascent reticulocyte (Sawada et al., 1989; Wickrema et al., 1994). Integral membrane proteins that are associated with the cytoskeleton, such as Glycophorin A (Lee et al., 2004), Band 3, RhAG, GPC; (Salomao et al., 2010) are thus predominantly found on the reticulocyte. Some proteins are specifically sorted to the pyrenocyte, such as the erythroblast macrophage protein (EMP), β1 integrin, Basignin (or CD147) and other adhesion molecules (Lee et al., 2004; Soni et al., 2006; Griffiths et al., 2012; Gautier et al., 2016). Distinct differences in the membrane composition of pyrenocytes and reticulocytes is likely to direct macrophages to phagocytose extruded nuclei. The mechanism of selective retention or exclusion of specific proteins within newly generated reticulocytes is still largely unknown but involve association with the erythroid specific Spectrin/Ankyrin cytoskeleton (Lee et al., 2004; Keerthivasan et al., 2011; Satchwell et al., 2016). During erythropoiesis the cytoskeleton is rearranged to facilitate large protein membrane networks that control the structural integrity of erythrocytes in the circulation through regulation of morphology by modulation of protein-cytoskeleton interactions or ion-transport (Bruce et al., 2009; van den Akker et al., 2010b; Satchwell et al., 2011). These networks can be roughly divided into an Ankyrin network and a junctional network [for review see (Bruce, 2008; van den Akker et al., 2010c)]. Mutations within proteins that comprise these complexes results in an array of erythrocyte specific diseases like hereditary spherocytosis and elliptocytosis [reviewed in (Da Costa et al., 2013; Gallagher, 2013)]. It has been proposed that inclusion of proteins within such networks facilitates reticulocyte retention during enucleation and vice versa localization to the nuclei upon non-inclusion (Satchwell et al., 2016). For instance GPC is located to the pyrenocyte in 4.1 deficient erythroblasts while this protein normally is sorted to reticulocytes, and GPA and Rh-associated antigens are misdirected to the pyrenocyte in ankyrin deficient erythroblasts (Chasis et al., 1993; Satchwell et al., 2016). Aberrant sorting of proteins during enucleation may lay fundament to the development of pathologies like Hereditary spherocytosis and elliptocytosis. Mutations within membrane proteins or cytoskeleton proteins resulting in disturbed/weakened association between specific membrane proteins with the cytoskeleton lead to aberrant distribution to pyrenocytes (Salomao et al., 2010; Satchwell et al., 2016). A strict hierarchy is present that results from inter-dependency of specific membrane protein expression. For instance, loss of RhAG will lead to a complete loss of Rh proteins, a loss of Kell means a loss of Xk, and loss of RhCE or protein 4.2 leads to a significantly reduced level of CD47 (Cherif-Zahar et al., 1996; Rivera et al., 2013; Mordue et al., 2017). Thus mutations within such “master” proteins may have significant domino effects on other proteins and thus the constitution and functionality of these large membrane complexes affecting erythrocyte stability. However, for proteins that are not part of these cytoskeleton-membrane protein hubs it is unknown if underlying mechanisms are specific or not. More work is required in order to determine the exact mechanism of protein sorting during enucleation. Understanding the nature of the sorting process can shed some light on the protein loss combined to the mutation in membrane-cytoskeleton complexes. This knowledge can aid in development of possible prevention and treatment of Hereditary spherocytosis and Elliptocytosis. In addition, direction of specific membrane proteins to these large membrane complexes may facilitate specific incorporation of therapeutic proteins to be used as delivery tools. A role for lipid composition to the sorting of proteins cannot be ruled out as it has been shown that lipid modifications and alterations during pathology associate with increased adhesion capabilities of erythrocytes (Hillery et al., 1996; Connor et al., 1997; Kuypers et al., 2007).

## Maturation of Reticulocytes in the Circulation

**FIGURE 2:**
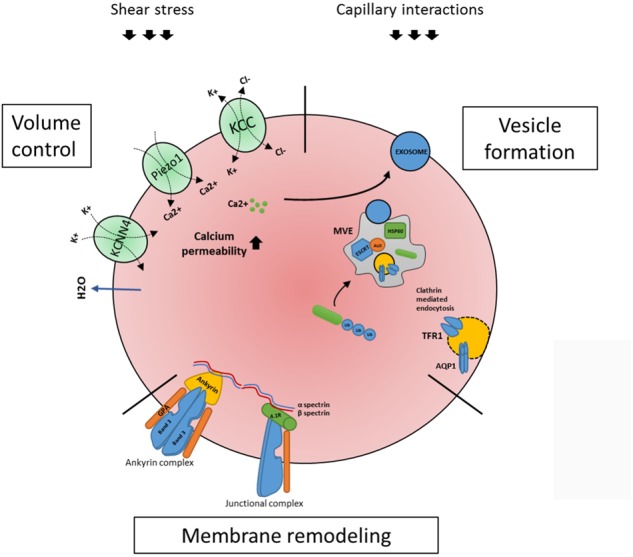
Processes occurring within the maturing circulating reticulocyte. Once enucleated, the newly formed reticulocyte migrates to the peripheral blood where it completes its maturation in a surrounding eliciting external forces, shear stress and capillary/splenic interactions. Three major events occurring are volume control, membrane remodeling and vesicularization. As for volume control, a key role is played by ion channels KCNN4 (or Gardos), Piezo1 and KCC1 (see text). During reticulocyte maturation an active membrane remodeling is also occurring, in which the two main red blood cells complexes, the ankyrin and the junctional complexes (linked through a cytoskeleton network) are involved (see text). Lastly, the reticulocyte makes use of the ESCRT- mediated multi vesicular body (MVB) machinery to expel unwanted proteins. Proteins, e.g., TFR1 and Aquaporin1 are ubiquitinated and routed to the MVB where the ESCRT complex (in particular its associated protein Alix and Hsp60) contributes in assembling the MVB; this process will result in exosome releasing, which is also described to be triggered by calcium influx (see text). All three processes are mechanistically linked (see text) and need to occur in order for a reticulocyte to mature to an erythrocyte.

Although the term reticulocytes generally refers to enucleated erythroid cells that are not yet fully biconcave, one must appreciate these reticulocytes are a heterogeneous population of all stages in between enucleated reticulocytes and erythrocytes (Malleret et al., 2013; Ovchynnikova et al., 2017). These stages are characterized by progressive loss of RNA and membrane proteins that are cleared during maturation, such as CD71 or CD49d. In addition, reticulocyte maturation can be defined by size, morphology, biomechanical properties and metabolic state (Malleret et al., 2013). Diversity within the reticulocyte population was first described in early 1930’s (Heilmeyer and Westhäuser, 1932). Based on brilliant Cresyl blue staining patterns, four stages were identified in the circulation: group I, group II, group III, and group IV (Heilmeyer and Westhäuser, 1932). Implementation of flow cytometry methods and use of Thiazole Orange (TO) to visualize RNA provided a more advanced reticulocyte maturity index classification system (Davis et al., 1995; Choi and Pai, 2001; Wollmann et al., 2014). This system, however, is proven to be inaccurate in certain conditions. Presence of hemoparasites and pathological erythrocyte inclusions such as Howell–Jolly bodies may result in false positive staining. Gradual loss of the transferrin receptor makes it an informative marker for reticulocyte maturation (Malleret et al., 2013). Based on CD71 staining of cord blood derived reticulocytes Malleret et al. (2013) described four stages of reticulocyte maturity namely CD71high, CD71medium, CD71low and CD71negative. Utilization of TO staining together with CD71 provides a more accurate distinction of various stages of reticulocyte maturation, mostly due to the fact that CD71 loss occurs prior to the loss of RNA. Based on CD71/TO dual staining it is possible to differentiate four stages of reticulocyte maturation from early reticulocytes R1 to late reticulocytes R4 (Malleret et al., 2013; Ovchynnikova et al., 2017). Upon release from the erythroblastic island, reticulocytes are irregularly shaped, multilobular cells that are far from being flexible compared to mature red blood cell (Mel et al., 1977; Malleret et al., 2013). To adopt a bi-concave morphology, stability, and the ability to deform these cells have to undergo a maturation process. Membrane-cytoskeleton rearrangement may be an important step allowing the transition from an unstructured reticulocyte to a morphologically biconcave and functional erythrocyte (Waugh et al., 1997, 2001). It takes 1–2 days in the circulation for the reticulocyte to obtain bi-concaveness and mature into fully functional erythrocytes (Chasis et al., 1989). Surprisingly, mature reticulocytes are stable and deformable as mature erythrocytes, while in young reticulocytes mechanical stability and membrane deformability are decreased (Chasis et al., 1989; Waugh et al., 1997). In addition, extracellular epitope exposure of specific membrane proteins is changing during reticulocyte maturation (Malleret et al., 2013; Ovchynnikova et al., 2017). This most likely involves specific protein reorganization into large protein complexes masking specific epitopes but could also suggest structural protein remodeling due through specific interactions or through lipid content remodeling (Sailaja et al., 2004; Ovchynnikova et al., 2017). Unfortunately, not much is known about how volume loss and discoid shaping are manifesting during differentiation, and what is known is mainly obtained from animals with reticulocytosis caused by stress erythropoiesis. Generalizing, there are three major events occurring in this time window: volume control, membrane remodeling and vesicularization (**Figure 2**).

### Reticulocyte Volume Control

It has been calculated that the average cell volume of a reticulocyte once egressed from the bone marrow is 115 fL and decreases to 85 fL in mature erythrocytes, while the hemoglobin concentration (CHC) is increasing from 27 to 33 g/dL (Gallagher, 2017). Reticulocyte volume loss can be attributed to two processes, (i) through loss of membranes and intracellular remnants due to exosome/vesicle formation/shedding and (ii) through actions of transporters that regulate the cation/anion and water content and thereby cell volume. The contribution of each process to the eventual 20% loss of cell volume is not known. The potassium chloride cotransporter (KCC) is important in these processes. When KCC is activated, the cell loses potassium, chloride and water leading to cell volume loss. It was demonstrated that KCC activity decreases during reticulocyte maturation (Canessa et al., 1987; Bize et al., 2003; Quarmyne et al., 2011). It was also proven that Sickle Cell reticulocytes have an altered regulation of KCC activity, possibly being the cause of Sickle Cells dehydration. In conjunction with KCC, the mechanosensitive channel Piezo1 plays a role in volume regulation (Bagriantsev et al., 2014). When activated, Piezo1 causes Ca^2+^ to enter the cell, leading to the activation of the Gardos-channel associated with a change on K+ and Cl- influx and water loss, essential events that allow the red blood cell to narrow through the blood capillaries (Fermo et al., 2017). Presently, numerous gain of function mutations in the mechanosensor Piezo1 have been found that cause the most common form of hereditary stomatocytosis namely hereditary xerocytosis (Zarychanski et al., 2012; Albuisson et al., 2013; Andolfo et al., 2013; Da Costa et al., 2013; Archer et al., 2014; Beneteau et al., 2014; Sandberg et al., 2014). Interestingly, a Piezo channel agonist independent of the mechanosensitive mode of activation has been found, YODA1, that selectively activates the Piezo channel function. This induces calcium influx and erythrocyte dehydration confirming its the role of Piezo-1 in volume control (Cahalan et al., 2015; Syeda et al., 2015). Calcium permeability in reticulocytes is significantly increased compared to erythrocytes. Healthy reticulocytes are 43 times more permeable to Ca^2+^ compared to mature erythrocytes (Wiley and Shaller, 1977), which may be linked to mechanosensing, through, e.g., Piezo1. Indeed, inhibiting *N*-methyl-D-aspartate-induced calcium influx during erythroid maturation results in defective differentiation (Makhro et al., 2013). In addition, increased intracellular calcium levels due to secondary calcium-dependent phosphorylation processes have been observed in reticulocytes and may possibly link to specific phosphorylation of junctional and ankyrin complex proteins necessary for erythrocyte structural integrity (de Haro et al., 1985; Liu et al., 2010). Not all signal transduction that results in calcium influx in erythrocytes is functional in reticulocytes indicating an interesting signal specificity. For instance, lysophosphatidic acid (LPA) stimulation of RBCs is known to increase Ca^2+^ influx in erythrocytes (Yang et al., 2000), however, reticulocytes lack a Ca^2+^ response to LPA stimulation (Wang et al., 2013). Interestingly, Ca^2+^ ionophore-induced laminin association in SCD erythrocytes was not observed in reticulocytes (Burnett et al., 2004). It is known that increased Ca^2+^ flux induces release of vesicles in erythrocytes, which can contribute to the vast membrane loss during reticulocyte maturation (Kostova et al., 2015). How calcium signaling contributes to reticulocyte maturation will need to be further investigated, but reticulocytes express several surface molecules that induce Ca^2+^ fluxes.

### Extracellular Factors Involved in Reticulocyte Maturation

Exiting the bone marrow, reticulocytes end up in a complex environment where their fate and development may be determined by a plethora of factors such as interactions with other blood components, endothelial cells, red pulp macrophages of the spleen, macrophages in liver and physical forces such as shear stress and osmolarity changes. Whilst the role of splenic and liver interactions in the development of reticulocyte was extensively studied (Yuditskaya et al., 2010; Klei et al., 2017), the influence of the shear stress forces and capillary interactions remains to date unclear. Reticulocytes share characteristics with erythrocytes of sickle cell disease (SCD) patients. Therefore, the physiology of sickle cells and their interaction with endothelium can shed some light on the fate of healthy reticulocytes in the blood stream. Patients with SCD are prone to develop painful vaso-occlusive crisis, which is characterized by abnormal interaction between red cells, leukocytes and endothelium and can lead to aggregate formation (Manodori et al., 1998). Reticulocyte-specific maintenance expression of adhesion proteins that may facilitate interaction with the endothelium. Among those proteins are α_4_β_1_ integrin which is widely expressed on reticulocytes and interacts with VCAM-1, CD47 which binds to thrombospondin and Lu/BCAM which interacts with laminin (Gee and Platt, 1995; Udani et al., 1998). Hillery et al. (1996) showed that during reticulocytosis an increase, albeit modest, in thrombospondin association was observed, which may indicate increased endothelial interaction of reticulocytes. Although interactions with the endothelium would generally be considered as unwanted, it is unknown why reticulocytes still express specific receptors that could facilitate these interactions and if a so whether biological relevance can be found concerning endothelial interactions. Sickle cells display a higher activation status of Lutheran while healthy reticulocytes display higher expression of Lu/BCAM compared to erythrocytes, while the activation status of Lutheran in reticulocytes is less clear (Malleret et al., 2013). Binding of sickle cells to laminin is mediated via stimulation of β-adrenergic receptor and subsequent activation of G_αs_, which stimulates adenylyl cyclase. This enzyme induces conversion of ATP to cAMP, which activates PKA. This kinase mediates Lu/BCAM adhesion to laminin via yet unknown targets (Hines et al., 2003). Another molecule which is upregulated on sickle cells as well as on healthy reticulocytes is CD47, which binds to soluble and surface associated thrombospondin. Brittain et al. (2001) demonstrated increased affinity of sickle cells to thrombospondin upon shear stress application via activation of G_1_ tyrosine kinase pathway. The relations between maturing red blood cells and macrophages of spleen and liver is important. It is proposed that macrophages of red pulp take part in “polishing” of the red cell membrane by removing excessive membranes and exosomes [reviewed by (Klei et al., 2017)]. Indicative of this “polishing” role is the presence of exosomes and inclusions such as Howell–Jolly bodies in splenectomized patients, whereas in healthy patients less than 2% of red blood cells have visible inclusions. Splenectomized patients show an increase in reticulocyte numbers. It has been speculated that this is due to delayed maturation and not by increased erythroid flux (De Haan et al., 1988). General reticulocyte parameters like morphological changes or volume loss are, however, not affected upon splenectomy indicating that spleen macrophages are not essential for these maturation parameters. Despite the specific quality control functions by splenic macrophages, reticulocyte maturation proceeds as normal in splenectomized patient indicating that reticulocyte maturation is partly intrinsic, or dependent on passage through other erythroid-regulating organs such as the liver, and may be promoted by other processes such as shear stress-induced signaling or endothelial interactions.

### Membrane and Cytoskeleton Remodeling During Reticulocyte Maturation

Reticulocyte maturation is accompanied not only by a loss of cytosolic organelles, but also by an intense membrane remodeling. In red blood cells, two major membrane complexes are found: the ankyrin complex and the junctional (or 4.1 based) complex. These complexes are linked through a fine cytoskeletal network which involves spectrin as the main constituent. The anion transporter protein Band 3 (Anion Exchanger 1) plays a key role, as it is the main component of both complexes and anchors them to the cytoskeleton (Mohandas and Gallagher, 2008; van den Akker et al., 2010c). The cytoskeletal network comprises proteins like α-spectrin, β-spectrin, actin, protein 4.1R, ankyrin R, protein 4.2, p55, adducin, dematin, tropomyosin, and tropomodulin (Liu et al., 2011). Ankyrin deficiency causes a disruption of the membrane network, including the incapacity of Band 3 to form tetrameric complex and degradation of protein 4.2, together with loss of reticulocytes protein due to increased sorting of these proteins in the pyrenocyte (Satchwell et al., 2016). Some of these proteins are also lost during normal reticulocyte maturation, such as cytosolic actin and tubulin while some non-cytoskeletal proteins including GLUT4, Na^+^K^+^ ATPase, GPA and CD47 are only reduced (Liu et al., 2010). The reduction involves also the membrane protein Glycophorin A (GPA), although in our recent study we showed that during reticulocyte maturation GPA expression is only changing slightly compared to mature erythrocytes (Ovchynnikova et al., 2017). Band 3, Rh, RhAG, GPC, and XK are increased, most probably due to a loss of the membrane surface (Liu et al., 2010). The mechanisms behind the differential change in expression of the membrane proteins are nowadays still unknown. The loss of tubulin and actin has been proved to be mediated by the ubiquitin-proteasome system (Liu et al., 2010). Phosphorylation of certain proteins could play a role. Indeed, phosphorylation of 4.1 protein is significantly higher in reticulocytes compared to erythrocytes (Liu et al., 2010). Phosphorylation of protein 4.1 regulates the strength of the spectrin-p55-4.1R ternary complex, and increased phosphorylation in reticulocytes weakens the association of these proteins in reticulocytes compared to erythrocytes (Manno et al., 2005). Decreasing 4.1 phosphorylation during reticulocyte maturation will strengthen the junctional complex and regulates the mechanical stress mature erythrocytes can endure. Interestingly, a spectrin fragment mimicking a β spectrin region involved in the ternary complex formation is incorporated more easily in reticulocytes than erythrocytes, resulting in a more unstable junctional complex in reticulocytes (Manno et al., 2005). Interestingly other proteins comprising the junctional complex, GPC, XK, and Kell, are more extractable upon detergent treatment underscoring the weakened link of these membrane proteins to the underlying spectrin cytoskeleton. In contrast, proteins within the ankyrin complex remain bound to cytoskeleton (band 3, Rh, RhAG, and GPA), suggesting that during reticulocyte maturation the latter complex is already formed, while the former is still remodeling, eventually contributing to a more stable cell with an higher tolerance toward shear stress. Presently little is known about the regulation of these complexes through phosphorylation in reticulocytes. The assembly of these complexes during erythropoiesis is critical to the eventual stability of *in vitro* produced erythrocytes. Indeed, due to the optimization of *in vitro* human and mouse erythroblast culture systems, we are beginning to understand the assembly of these large protein complexes during erythropoiesis (Tchernia et al., 1981; Hanspal et al., 1992, 1998a; van den Akker et al., 2010b; Satchwell et al., 2011; Trakarnsanga et al., 2017).

### Protein Removal During Reticulocyte Maturation Through Exosome Shedding

Another example of membrane remodeling involves the transferrin receptor TFR1 (also referred as CD71). In general, iron-loaded transferrin binds to TFR1 and internalizes via clathrin-mediated endocytosis. In the endosome the transferrin-receptor is stripped of the iron and transferrin-receptor complex returns to the membrane, where TFR1 release apo-transferrin and with that finishes the cycle (Hentze et al., 2010). But how is TFR1 lost in reticulocytes? As heme production and transcription of globins is shut down due to mitochondrial loss and enucleation, respectively, there is no need to import the oxidative-damage-inducing iron anymore. Thus, the nascent reticulocyte has incorporated all the iron needed, and the transferrin receptor must be removed, in order to prevent overloading of iron. This involves the switching from recycling of the receptor to the encapsulation into vesicles that will be removed. It is known since 1983 that the transferrin receptor sheds into vesicles. In fact, the term exosome or vesicle release has been shown for the first time in reticulocytes (Johnstone et al., 1987), but it was only later that these vesicles were defined as exosomes. This receptor is sorted through the multivesicular endosomes (MVEs) pathway, that terminates with the incorporation of TFR1 in a new formed vesicle that is subsequently fused with plasma membrane in order for the content to be released into the extracellular medium (Pan et al., 1985). About the molecular players involved in this process, it has been shown that hsp60 and the ESCRT associated protein Alix co-localize with TFR1 in reticulocytes exosomes, confirming the involvement of the endosomal – vesicular pathway in exosomes formation (Geminard et al., 2004). Interestingly, CD71 shedding can also be regulated in Ca^2+^ dependent manner and activated by extracellular transferrin (Savina et al., 2003). Among proteins that are cleared from reticulocytes through formation of vesicles is Aquaporin 1 (AQP1). This water channel is responsible for keeping a proper plasma membrane tonicity, in response to different osmotic perturbations. It co-localizes with TFR1 in the plasma membrane and in reticulocytes endosomal compartments (Blanc et al., 2009). Together with the knowledge that the loss of aquaporin-1 has been described as a prototype example of degradation through exosomes, recent data show that AQP1 is expressed at higher levels in adult red blood cell compared to cord blood cells, as well as in adult reticulocytes compared to cord reticulocytes. Such a difference possibly reflects a different gas exchange regulation in adult and neonatal red blood cells, although this remains to be confirmed (Schutte et al., 2016). Together with TFR1 and aquaporin 1, multiple proteins need to be disposed from the plasma membrane or the cytosol during reticulocyte maturation, presumably and sensibly from an evolutionary point of view via the same mechanism. The major process involves different routes of targeting proteins to multi-vesicular bodies (MVB), including the lipid domain inclusion, cytosolic inclusion and lectin mediated inclusion, targeting ubiquitinated, HSC/HSP bounds proteins and glycosylated proteins to MVBs (Guinez et al., 2007; Carayon et al., 2011). The ESCRT (Endosomal Sorting Complex Required for Transport) machinery comprises four complexes: ESCRT 0, I, II, III that are involved in MVB formation. Briefly, in the endosomal lumen, ESCRT 0, which has multiple ubiquitin binding domains, binds the ubiquitinated cargo (i.e., proteins destined to be included in the vesicles) and engages the other ESCRT subunits, while facilitating interactions with clathrin domains. ESCRT I and II are recruited and cause the deforming and involution of the membrane. The process is terminated by recruitment of ESCRT III by simultaneously pulling the cargo into the invagination and generating the MVB. It is generally believed that ESCRT complexes recognize ubiquitinated membrane proteins that need to be sorted into MVB (Odorizzi et al., 1998; Raiborg and Stenmark, 2009; Hislop and von Zastrow, 2011). In contrast, intracellular proteins may be routed to MVB via HSP70/HSC70 via ill-defined mechanisms. These mechanisms include, (i) HSC70 interacting with the endosomal membrane via electrostatic associations using its C-terminal basic region (Sahu et al., 2011), (ii) interactions with the ESCRT complex again via ubiquitin-modified targets (Dajani et al., 2001) or (iii) through ubiquitination of hsc70 itself (Pridgeon et al., 2009). Hsc70-dependent microautophagic processes are thus probably linked to MVB targeting and removal of unwanted proteins. Recently, UBE2O, an E2-E3 hybrid enzyme, which acts both as ubiquitin-conjugating enzyme and ubiquitin ligase has been found to play a key role in erythroid proteome remodeling among which ribosome elimination (Nguyen et al., 2017). A further link between autophagy and plasma-membrane vesicles was found with the co-localization of glycophorin A and the autophagy protein LC3 on intracellular reticulocyte vesicles (Griffiths et al., 2012). Griffiths et al propose that the final remnants of the plasma membrane are removed via an autophagosome/endosome hybrid compartment. Exosome composition indeed changes in protein composition during maturation (Carayon et al., 2011). Whether these vesicles are different entities or similar remains to be investigated as in other cell types the autophagosome can fuse with endocytic structures such as MVBs to generate an amphisome (Berg et al., 1998). Furthermore, efficient autophagic degradation requires functional MVBs and their fusion is again calcium dependent (Fader and Colombo, 2009). Normally, besides exosome release, these vesicle bodies are destined to fuse with the lysosome, causing the degradation of specific proteins (Saksena et al., 2007). However, in maturing reticulocytes these vesicles are exclusively re-routed to a secretory pathway (Pan et al., 1985; Blanc et al., 2009). The mechanisms and in particular the pathways involved in the formation and fusion of the vesicles with the membrane and consequential secretion are not yet fully understood. In the erythroleukemia cell line K562, exosome secretion is caused by an intracellular Ca^2+^ influx (Savina et al., 2003). Indeed, significant vesicle formation can be induced upon treatment of erythrocytes with the calcium ionophore ionomycin or through PKC activation (Kostova et al., 2015; Nguyen et al., 2016). Moreover, docking and fusion of MVB to the plasma membrane is regulated by calcium dependent small GTPase Rab11 (Savina et al., 2005). Of note, Rab11 has been shown to associate with the pericentriolar recycling endosome, as well as being involved in the regulation of transferrin recycling in the endosome (Ullrich et al., 1996). It is tempting to speculate that during reticulocyte maturation, routing of non-wanted proteins utilizes the same mechanism through which TFR1 is removed. The role of calcium signaling in MVB formation and fusion as well as the increased permeability of reticulocyte to calcium suggest a prominent but ill-characterized role for calcium signaling during reticulocyte formation. The formation, regulation and contents of MVB during reticulocyte maturation requires more attention as (i) it may provide key improvement to the *in vitro* culture of mature erythrocytes rather than the reticulocytes that are currently the end-stage of most culture protocols and (ii) specific pathologies may already manifest and initiate in the reticulocyte stage due to malfunctions within the sorting/secretory machinery. For instance, in dominant inherited beta-thalassemia’s, a heterozygous mutation in the beta globin chain results in unstable hemoglobins and rapid degradation of the beta chains, which may overload the available chaperone/sorting system resulting in additional secondary defects. In addition, an array of erythrocyte diseases is characterized by aberrant expression of membrane proteins that are normally completely or partially removed during erythropoiesis (e.g., BCAM, LW or CD44). However, specificity for over-expression of a small selection of membrane proteins is not easy to explain via the general mechanism of MVB-mediated protein removal. Although lists of proteins identified in reticulocyte exosomes have been published (Carayon et al., 2011), more in-depth research into the extruded but also the intracellular vesicles bodies may uncover much needed insight into the cargo and may identify crucial proteins that play a role in the formation and secretion machinery. The fate of exosomes is interesting from a signaling and immunogenic point of view. The membrane of reticulocyte exosomes contains 20% exposed Phosphatidylserine (PS) on the outer leaflet, which is a potent phagocytic trigger for many cells including cells that are in close contact with reticulocytes, e.g., endothelial cells, macrophages and neutrophils (Vidal et al., 1989). This provides a safe clearance mechanism; however, these vesicles may also function as conveyors of specific signals upon uptake by target cells. Research on this topic and specifically concerning reticulocyte exosomes is lacking.

## Conclusion

In the bone marrow erythropoiesis progresses until the enucleated reticulocyte stage, after which the reticulocytes are released into the circulation where final maturation occurs. Is there an evolutionary benefit for this maturation to occur in the circulation and not within the bone marrow? Early reticulocytes seem non-optimally adapted to the shear stress within the circulation and display lower deformability. Despite this, it also becomes increasingly clear that reticulocyte maturation is an interplay between intrinsic processes and extrinsic processes, including exosome formation, interactions with splenic and liver cells (e.g., macrophages) and circulation-induced shear forces. Interestingly, erythroid culture protocols are mainly driven by the intrinsic ability of erythroblasts to differentiate into reticulocytes but fail to produce fully biconcave erythrocytes, stressing that specific maturation cues are missing. Although, these missing signals may partly originate from the bone marrow niche, it is surprising that pure >95% enucleated reticulocytes can be cultured without this support (Miharada et al., 2006; van den Akker et al., 2010a; Giarratana et al., 2011). Failure to progress from reticulocytes to fully biconcave erythrocytes thus suggests a lack of signals that may be partly independent and dependent of the bone marrow niche. Of note, also stromal co-cultures (MS5 or OP9) do not completely result in reticulocyte maturation to erythrocytes (Giarratana et al., 2005; Lu et al., 2008). Indeed, a recent study published a characterization of the secretory proteins from OP9, and such studies may eventually point to specific factors to will further facilitating erythropoiesis and possibly reticulocyte maturation (Trakarnsanga et al., 2018). One such extrinsic signal may be transport/exchange regulation of volume control upon circulation-induced shear stress to facilitate reticulocyte maturation. However, more research must be performed dissecting the contribution and connections between intrinsic processes and extrinsic factors to this maturation. Even prior to this, the field is currently still assessing the identity/nature of these external factors and their concomitant erythrocyte “signal transducer” counterparts before characterization and optimization can begin. For instance, the connection between exosome formation, cargo selection (e.g., ubiquitination) and cation/anion permeability/conductance is interesting to pursue. With the aid of novel techniques to separate and discriminate the various reticulocyte maturation stages and the progressive use of lower cell numbers in -omic approaches will facilitate this research. Future erythroid culture protocols will need to incorporate the regulation (activation) of these processes to ensure complete differentiation to fully biconcave erythrocytes. The promise of *in vitro* cultured patient catered personalized transfusion products as well as erythrocytes carrying specific cargo for therapeutic purposes are potent drivers that justify research into this last elusive step of erythropoiesis.

## Author Contributions

EO, FA, MvL, and EA wrote the manuscript. The manuscript was critically revised by all authors.

## Conflict of Interest Statement

The authors declare that the research was conducted in the absence of any commercial or financial relationships that could be construed as a potential conflict of interest.
